# Study on the impact of digital countryside construction on fostering sports industry development: based on the moderating role of market-oriented factor allocation and the mediating role of rural consumption upgrade

**DOI:** 10.3389/frai.2025.1660947

**Published:** 2025-10-14

**Authors:** Xinxin Zhang, Sen Wang, Tianpei Li, Shuangcheng Sun

**Affiliations:** ^1^Myongji University, Yongin, Republic of Korea; ^2^Zhengzhou Zhengdongxinqu Foreign Language School, Zhengzhou, China; ^3^Jeonbuk National University, Jeonju, Republic of Korea; ^4^Anhui Institute of Information Technology, Wuhu, China

**Keywords:** digital countryside construction, sports industry, market-oriented factor allocation, rural consumption upgrade, structural equation model

## Abstract

**Introduction:**

Against the backdrop of China’s coordinated advancement of the “Digital China” and “Rural Revitalization” development strategies, digital countryside construction has emerged as a new opportunity for the sports industry.

**Methods:**

Based on panel data from 21 provinces in China from 2015 to 2023, this study employs fixed-panel models, moderation effects models, and mediation effects models to examine the relationship between digital countryside construction and the sports industry, as well as its underlying mechanisms.

**Results:**

Digital countryside construction exerts a significant positive promotion on the sports industry; Market-oriented factor allocation exerts a significant positive moderating influence on the impact of digital countryside construction on the sports industry; Rural consumption upgrade plays a significant positive mediating role in the influence of digital countryside construction on the sports industry. In terms of regional heterogeneity, the eastern region exhibits a significant promoting effect, while the central region shows a suppressing effect that is not statistically significant. The western region also demonstrates a promoting effect, though it is not statistically significant.

**Discussion:**

Implementing region-specific development strategies, tailored to local conditions, is of paramount significance for achieving the maximal policy benefits of digital countryside construction. This should be accomplished by deepening market-oriented reforms and enhancing digital infrastructure to foster balanced and high-quality development of the sports industry in both urban and rural areas.

## 1 Introduction

Currently, the widespread application of digital technology has become a key driver of high-quality economic and social development in rural areas. Digital countryside construction (DCC), a pivotal rural development strategy integral to the “digital China” and “rural revitalization” strategies, was initially outlined in the 2018 No. 1 Document, titled *“Opinions of the Central Committee of the Communist Party of China and the State Council on Implementing the Rural Revitalization Strategy.”* It has not only markedly enhanced rural informatization and intelligent infrastructure but has also significantly impacted industrial structure optimization, social governance innovation, and urban–rural integration (Lei et al., 2023).

The sports industry, a crucial component of the contemporary service sector, is essential for stimulating economic growth, enhancing public health, and boosting social and cultural vitality (You and Gao, 2024). It is closely intertwined with the objectives of rural revitalization—including advancements in public services, industrial development, talent cultivation, cultural heritage, ecological protection, and organizational capabilities—as highlighted by the General Administration of Sport of China in the *“Guiding Opinions on Promoting the Role of Sports in Rural Revitalization”* issued in June 2023.

In recent years, the deepening implementation of the rural revitalization strategy has accelerated the process of DCC. Concurrently, the integration of the sports industry through technologies such as big data, artificial intelligence (AI), and the Internet of Things (IoT), creating novel drivers for the development of the rural economy (Cheng and Wang, 2024). Specifically, the enhancement of digital infrastructure has notably improved public sports services in rural areas, thereby increasing the convenience and accessibility of sports participation for rural residents (Li and Gao, 2025). Furthermore, digital technology has facilitated the integrated development of the sports industry with other sectors such as rural tourism and elderly healthcare, thus expanding the sports consumption market (Liu, 2025). Considering this context, it is crucial to decipher the impact of DCC on the development of the sports industry and elucidate the underlying mechanisms that drive this impact. Herein, a benchmark regression model is employed, utilizing provincial-level panel data from China (2013–2022), and a fixed-effects model is incorporated to analyze the relationship between DCC and sports industry development. The primary objectives are twofold: (1) to quantitatively analyze the direct impact of DCC on the development of the sports industry, and (2) to explore the potential mechanisms through which DCC influences the sports industry. The findings will provide valuable theoretical and practical insights into leveraging digital strategies to foster the sustainable development of the rural sports industry, thereby enriching existing literature in the fields of digital economy and sports industry development.

### 1.1 Literature review

The concept of the *digital countryside* was first introduced in the *Strategic Plan for Rural Revitalization (2018–2022)*. As a critical component of China’s national rural revitalization strategy, DCC aims to modernize agriculture and rural areas through the application of advanced information technologies, thereby facilitating integrated urban–rural development (Li et al., 2025). In recent years, a growing body of scholarly research has explored the connotations and extensions of DCC from manifold perspectives.

From an agricultural perspective, existing studies predominantly concentrate on themes including high-quality agricultural development, green transformation, and rural common prosperity. Wen (2025) posited that DCC served as a pivotal strategy to foster the high-quality development of the rural economy and to facilitate the deep integration of rural industries. Empirical analysis conducted by Yang et al. (2025) and colleagues demonstrated that DCC significantly fostered green agricultural development by reducing energy consumption, diminishing pollution emissions, and enhancing agricultural productivity. Similarly, Wang (2025) asserted that DCC was an essential strategy for achieving common prosperity in rural areas. Empowered by information technology, DCC is capable of optimizing agricultural production models, elevating farmers’ incomes, and narrowing the urban–rural development gap.

From a broader perspective, scholars have examined the impact of DCC on urban–rural integration and county-level economic development. Shen and Zheng (2024) found that DCC significantly promoted high-quality urban–rural integration, with market-oriented factor allocation (MFA) serving as a key moderating factor. Sun et al. (2025) concluded that within the expansive framework of “digital China,” DCC had altered the bias of technological progress in county economies—shifting toward capital-intensive development with an increasing degree of capital bias.

As an integral segment of the contemporary service economy, the sports industry possess substantial significance in fostering economic growth, enhancing public health, and enriching social and cultural life (Abrahamyan, 2023). In recent years, with the implementation of national policies supporting the sports industry, academic research has increasingly concentrated on its development, yielding a wealth of theoretical and empirical findings.

At the theoretical level, studies have explored foundational frameworks, functional mechanisms, and pathways for optimizing the development of the sports industry. Kang et al. (2024) emphasized that institutional infrastructure constituted the foundational framework for the effective functioning of the sports industry, arguing for the importance of top-level design in establishing a mutually reinforcing system. Zhang and Wang (2021) identified factor supply and market demand as the dual endogenous driving forces essential for the high-quality development of the sports industry. Xu et al. (2019) and colleagues believed that the essence of high-quality development of the sports industry lay in the expansion of the sports program sector, innovation in content, and the breaking of traditional development constraints.

At the empirical level, scholars have examined relevant themes such as industrial synergy and structural transformation. As the sports industry evolves toward greater integration and intelligence, new patterns have emerged. For example, Li et al. (2023) discovered that the integration of the digital economy and the sports industry demonstrated a strong and growing degree of coupling coordination, evolving from fragile to robust levels. Zheng et al. (2023) highlighted the challenges facing the development of AI-empowered sports, including the digital DCCide, limited mastery of core technologies, and insufficient high-level talent.

As a vital component of the rural revitalization strategy, DCC utilizes information technology to stimulate rural development by enhancing digital infrastructure and facilitating the digital transformation of agriculture. In this context, the sports industry contributes to upgrading rural industrial structures by fostering rural outdoor sports, folk sports events, and recreational programs, thereby transforming ecological resources into distinctive local industries.

On one hand, research on the integration of DCC with the sports sector remains limited. Liang (2024) demonstrated that DCC significantly promoted the development of rural sports tourism by analyzing its underlying mechanisms, with seven key mechanisms identified, including the construction of informatization infrastructure and the enhancement of digital services. Similarly, Gao et al. (2024) and colleagues posited that the theory of holistic governance accurately aligned with the value logic of providing public services for national fitness in rural areas.

On the other hand, in the realm of rural revitalization and the integration of the sports industry, researchers have uncovered a variety of valuable insights. Lu et al. (2024) and co-authors proposed that to address the current challenges faced by the rural sports industry, emphasis should be placed on ecological transformation, innovation in resource structures, and enhancing resilience for high-quality development. Wang (2025) and colleagues discovered a significant positive spatial correlation between the coupling coordination level of the sports industry and rural revitalization, exhibiting clear spatial clustering characteristics.

Nevertheless, a significant academic gap persists regarding the intersection of DCC and sports integration. The present study aims to explore the mechanisms through which DCC influences the development of the sports industry, which would offer meaningful scholarly value.

Collectively, existing research primarily investigated the relationship between digital technology and industrial development from two key perspectives: Firstly, studies on DCC have employed quantitative methods to verify its macro-level contributions to rural economic growth and household consumption. Secondly, research on the sports industry has predominantly concentrated on the impact of specific factors—such as the digital economy and digital finance—on the transformation and upgrading of the sector. However, despite these studies offering important theoretical foundations, a significant research gap remains: few have systematically examined how comprehensive DCC—as a holistic strategy—affects the development of the rural sports industry through specific mechanisms. This study seeks to bridge the existing gap by developing an empirical model to elucidate the intrinsic causal relationship between DCC and the sports industry.

Compared to previous studies, the contributions of the present study are threefold: (1) it examines the theoretical relationship between DCC and the development of the sports industry, thus expanding the literature in this field; (2) it incorporates MFA and rural consumption upgrade (RCU) as mediating and moderating variables to explore the inherent mechanisms using appropriate econometric models, thereby contributing novel perspectives to the development of the sports industry; and (3) it investigates regional heterogeneity to assess inter-city differences in the relationship between DCC and sports industry development, offering rational explanations based on regional disparities.

## 2 Theoretical analysis and research hypothesis

### 2.1 The impact of DCC on the development of the sports industry

As a key component of China’s rural revitalization strategy, the core objective of DCC is to drive the modernization and transformation of sectors such as agriculture, culture, education, and healthcare through the integration of digital technologies. In recent years, the Chinese government has introduced a series of supportive policies to facilitate DCC and encourage the application of digital technologies to upgrade rural industries.

In this context, the sports industry—an essential driver of rural economic development and a contributor to enhancing residents’ quality of life—has been significantly influenced by DCC. Specifically, DCC has enhanced the informatization level of rural sports infrastructure. Technologies such as IoT, big data, and AI have enabled intelligent management of rural sports facilities, thereby enhancing their utilization rates (Zhao X. et al., 2024; Zhao Y. et al., 2024). Systems for online reservations, intelligent monitoring, and digital operations have optimized the allocation of sports resources, reduced venue idleness, and reinforced the capacity of rural public sports services. Moreover, digital technologies have facilitated the market-oriented development of the rural sports industry. The emergence of e-commerce and short-video platforms has created new marketing and promotional avenues for rural sports tourism, training programs, and event hosting (Zhao X. et al., 2024; Zhao Y. et al., 2024). Through the integration of “Internet + Sports,” rural programs—including traditional ethnic sports, outdoor adventures, and grassroots competitions such as “Rural BA” and “Rural Super”—have been able to overcome geographical constraints and attract a extensive consumer base. These have contributed to the expansion of the rural sports tourism and event markets, extending the value chain of the sports industry (Tan and Bu, 2025).

Moreover, DCC has transformed the sports consumption behavior of rural residents. The prevalence of smart devices has facilitated convenient access to fitness content, while services such as remote coaching and online classes have increased sports participation. These changes have not only stimulated rural demand for sports consumption but also prompted the sports industry to optimize its supply structure to meet the DCCerse and personalized market needs (Zhang and Dong, 2024). Based on the above analysis, the following research hypothesis is proposed:

*H1*: DCC exerts a significant positive impact on the development of the sports industry.

### 2.2 The regulating role of market-oriented factor allocation (MFA)

Digital technologies have significantly enhanced the allocation efficiency of key production factors—such as capital, technology, and labor—by reshaping the pathways of factor circulation and redefining value creation models. They also assist in overcoming traditional barriers of time, space, and information asymmetry within factor markets (Zhang et al., 2025).

Firstly, in the context of DCC, technologies such as big data, remote sensing, and blockchain are applied to land information management systems, enhancing the scientific planning and utilization of rural sports infrastructure. This alleviates issues of idle land and inefficiency, optimizes the structure of land resource allocation, and revitalizes the market for land use in rural sports development (Fan and Kang, 2023).

Secondly, the rural sports industry has historically suffered from limited financing channels and long capital return cycles. The advent of digital finance has offered solutions by expanding funding sources. Progressive digital tools—such as inclusive finance, crowdfunding models, and blockchain-enabled risk control mechanisms based on big data—have provided the rural sports industry with increased social capital, improved capital allocation efficiency, and reduced investment risks, thereby fostering its market-oriented development (QI et al., 2020).

Thirdly, DCC has also promoted the optimal allocation of human resources within the sports industry. Supported by digital tools such as distance education and online training platforms, rural residents are able to access more professional instruction, which enhances the overall skill level of practitioners in the rural sports industry (Wang and Wu, 2024). Furthermore, the establishment of digital employment platforms helps to overcome geographic barriers, enabling professional sports talents to accurately meet rural demands, thus optimizing the human resources structure and spurring industry competitiveness. Based on the above analysis, the following hypothesis is proposed:

*H2*: MFA exerts a positive moderating effect on the relationship between DCC and the development of the sports industry.

### 2.3 The mediating role of rural consumption upgrade (RCU)

DCC serves not only as a strategic path for rural revitalization but also as a key catalyst for RCU (Wang et al., 2021). It enhances digital infrastructure, improves information flow, and raises residents’ income levels, thereby effectively stimulating the consumption demand and promoting the expansion and upgrade of the rural sports industry.

On one hand, advancements of information and communication technologies have narrowed the digital DCCide between urban and rural areas. Consequently, rural production and lifestyles have been significantly altered by enhanced information infrastructure, optimized factor allocation, and expanded agricultural product circulation channels. This, in turn, enhances the digital literacy and consumption capacity of farmers, stimulates rural domestic demand, and reshapes the rural consumption structure—from subsistence-level consumption to development-oriented and enjoyment-oriented consumption. These alterations offer robust technological and institutional support for RCU (Bi and Chu, 2025).

On the other hand, RCU is accompanied by increased health consciousness and transformed residential lifestyles, which results in a growing, DCCerse, and multi-level demand for sports consumption. The rising demand for physical fitness, leisure, and recreation services in rural areas has spurred the expansion of the sports industry, enriching its product offerings and services. Against the backdrop of urban–rural integration, this trend fosters the extension of sports resources to grassroots communities and hastens the optimization of the sports industry’s spatial distribution, service models, and value chains. Consequently, RCU has emerged as a crucial driver in fostering the coordinated development of regional economies and enhancing national physical fitness (Wan and Wang, 2024). Based on the above analysis, the following hypothesis is proposed:

*H3*: DCC indirectly fosters the development of the sports industry through RCU.

**Figure 1:**
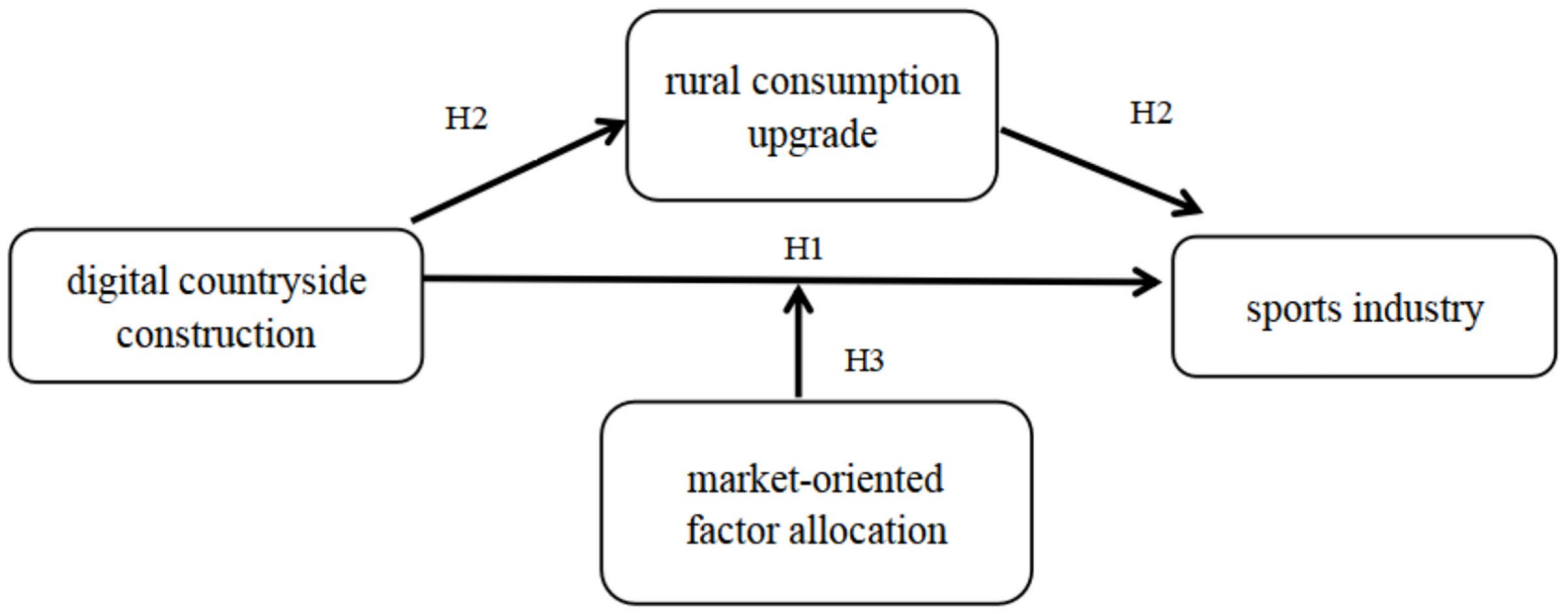
Path diagram of H1–H3 model.

In summary, according to the research hypothesis of this paper, the model path diagram of H1-H3 is constructed. As shown in Figure 1 below.

## 3 Research design

### 3.1 Variable selection

#### 3.1.1 Explained variables

The explained variable in this study is the sports industry (SIC). The sports industry is a comprehensive sector centered around sports activities, encompassing multiple domains such as events, services, manufacturing, and infrastructure. To quantify the level of sports industry development, this study draws on the evaluation index system proposed by Li et al. (2023), which assesses the industry across three dimensions: scale, structure, and efficiency (see Table 1).

**Table 1:** Evaluation system of sports industry development.

Primary indicator	Secondary indicator	Indicator description	Unit	Attribute
Industry scale	Total output of the sports industry	Total output value of sports production activities	100 million CNY	+
	Added value of the sports industry	Value added generated by sports production activities	100 million CNY	+
	Per capita sports venue area	Average sports venue area per person	m^2^/person	+
	Number of sports venues	Total number of sports venues	10,000 units	+
	Total employment in the sports industry	Number of employees in the sports industry	10,000 units	+
Industry structure	Proportion of sports industry in GDP	Proportion of sports industry added value in GDP	%	+
	Proportion of added value in GDP	Proportion of sports industry added value in GDP	%	+
	Fixed asset investment proportion	Proportion of sports fixed asset investment in total society	%	+
	Per capita sports consumption level	Sports consumption per capita as a share of disposable income	%	+
Industry efficiency	Added value growth rate	Added Value Growth Rate	%	+
	Annual growth rate of added value	Annual growth rate of value added compared to previous year	%	+
	Profit margin of the sports industry	Profit Margin of the Sports Industry	%	+
	Labor productivity	Value added per employee in the sports industry	10,000 CNY/person	+

Industry Scale: Refers to the overall volume and total output of the sports industry. Following the methodologies of Li et al. (2020), Kang (2022), and Li et al. (2023), this study selects six secondary indicators to represent the scale dimension. Industry Structure: Represents the proportion and distribution of various sectors within the sports industry, reflecting its internal composition and development trends. In line with Su et al. (2024) and Xu (2021), the structure dimension is measured using four secondary indicators. Industry Efficiency: Refers to the effectiveness of resource allocation in maximizing output and value under limited resources. This study adopts the evaluation frameworks of Kang et al. (2024) and Wang and Man (2020), using four secondary indicators for this dimension.

The data was normalized prior to model construction. Following Ren (2024), the entropy value method and a comprehensive evaluation model were utilized to measure the levels of development in the sports industry, yielding objective weights of the indicators.

The specific steps are see Equations 1–6.

Step 1: Normalize all the indicators using the following formula:


(1) $$z_{i}=\{\begin{array}{c} \frac{x_{i}-b}{a-b}+0.001,\text{for positive indicators}\\ \frac{a-x_{i}}{a-b}+0.001,\text{for negative indicators} \end{array}$$

where i(i=1,2,⋯,n) denotes the index of the indicators, *z_i_* denotes the normalized value of the i-th indicator, *x_i_* denotes the original value of the i-th indicator, *a* denotes the minimum value of all indicators, and *b* denotes the minumum value of all indicators.

Step 2: Calculate the ratio of the i-th indicator:


(2) $$p_{i}=z_{i}/\sum_{i=1}^{n}z_{i}$$

Step 3: Calculate the entropy of the i-th indicator:


(3) $$e_{i}=-\frac{1}{\ln N}\sum_{i=1}^{n}p_{i}\ln p_{i}$$

where *N* represents the time span. According to the selected cross-sectional data of 2015, N=8 .

Step 4: Calculate the variation coefficient of the i-th indicator:


(4) $$g_{i}=1-e_{i}$$

Step 5: Calculate the weight of the i-th indicator using the following formula:


(5) $$w_{i}=g_{i}/\sum_{i=1}^{n}g_{i}$$

where *g_i_* is the weight of the j-th indicator.

Step 6: Calculate the comprehensive development index of the sports industry, which is expressed as:


(6) $$u=\sum_{i=1}^{n}w_{i}z_{i}$$

where $\sum_{i=1}^{n}w_{i}=1$ .

Based on the above formula, the comprehensive development index of the sports industry for each province is calculated as shown in the Table 2 below (taking the years 2015 and 2016 as examples).

**Table 2:** Sports industry development index (for the years 2015 and 2016).

Region	Province	Sports industry development index	
		2015	2016
Eastern Region	Beijing	0.2355	0.2532
	Tianjin	0.1125	0.1084
	Hebei	0.1615	0.1520
	Liaoning	0.1927	0.1633
	Shanghai	0.2170	0.2402
	Jiangsu	0.6856	0.5027
	Zhejiang	0.2645	0.2411
	Fujian	0.1456	0.1294
	Shandong	0.3664	0.3793
	Guangdong	0.4028	0.4523
Central Region	Shanxi	0.0685	0.0730
	Inner Mongolia	0.0535	0.0494
	Anhui	0.2204	0.2276
	Jiangxi	0.0722	0.0592
	Henan	0.2882	0.2867
	Hubei	0.1918	0.2149
	Hunan	0.1159	0.0982
Western Region	Chongqing	0.0743	0.0689
	Sichuan	0.1909	0.1880
	Guizhou	0.0349	0.0505
	Yunnan	0.0465	0.0548

#### 3.1.2 Core explanatory variable: digital countryside construction(DCC)

The core explanatory variable is DCC, which refers to a systematic development strategy that utilizes modern information technologies—such as big data, the Internet, and AI—to drive rural revitalization by bridging the digital divide between urban and rural areas and enhancing digital infrastructure, services, and industries. Herein, the measurement framework developed by Yu et al. (2025) and others was employed, where DCC was evaluated across four dimensions: digital capital investment, digital information infrastructure, digital service level, and digital industry development. All indicators within these dimensions are endowed with appropriate weights using the entropy method to ensure objective evaluation.

#### 3.1.3 Moderating variable: market-oriented factor allocation(MFA)

The moderating variable is MFA, which represents the process by which capital, labor, and technology are allocated through market mechanisms, allowing production factors to flow efficiently to areas with the highest marginal productivity. It enhances resource optimization, stimulates economic vitality, supports high-quality industrial development, and reflects a transition from direct government intervention to institutional regulation and environment-building. Herein, the MFA measuring approach proposed by Marin (2024) was utilized, which encompassed three dimensions: labor market allocation, capital market allocation, and technology market allocation. The entropy method was used to derive appropriate weights for these indicators.

#### 3.1.4 Mediating variable: rural consumption upgrade (RCU)

The mediating variable is RCU, which reflects the transformation in rural residents’ consumption behavior in response to elevated income levels and improved quality-of-life aspirations. As the disposable income of rural residents increases, their consumption patterns have shifted from satisfying fundamental necessities to purchasing developmental, recreational, and health-related goods and services. Following Yang et al. (2025), the per capita consumption expenditure of rural residents was used as a proxy indicator for RCU.

#### 3.1.5 Control variables

To ensure a more accurate assessment of the relationship between DCC and the development of the sports industry, control variables should be introduced to account for confounding factors that may exert potential impact. Building upon the research endeavors of previous scholars, five control variables that may affect the development of the sports industry were identified: (1) Government intervention (Gov), as adopted by Bidayu (2025), is represented by the ratio of general public budget revenue to general public expenditure; (2) The level of financial development (Fin), as used by Yang et al. (2025) and others, is measured as the ratio of total deposits and loans of regional financial institutions to the region’s gross domestic product (GDP); (3) The investment capacity of rural households (Fic), as referenced by Yu, (2024), is measured by per capita fixed asset investment by rural households; (4) The governance level of rural roads (Roa), as utilized by Tian et al. (2024), is calculated as ((\text{total road mileage} − \text{highway mileage} − \text{first-class highway mileage}) / \text{provincial land area}) × 100; (5) Education level (Edu), as employed by Liu (2025), is measured by the number of high school students per 100,000 people.

#### 3.1.6 Data sources

Given the late development of the sports industry in certain provinces and incomplete data availability, most provincial-level administrative regions only began publishing official statistics in 2015. To ensure data authenticity, scientific reliability, and quality control, these provinces and corresponding years were excluded from the analysis. This study selects 21 provincial-level administrative regions in China (Beijing, Tianjin, Hebei, Shanxi, Inner Mongolia, Liaoning, Shanghai, Jiangsu, Zhejiang, Anhui, Fujian, Jiangxi, Shandong, Henan, Hubei, Hunan, Guangdong, Chongqing, Sichuan, Guizhou, and Yunnan) for panel data analysis spanning 2015–2023. The dataset comprises 189 samples, with missing values addressed through interpolation. Data were sourced from the Sports Bureaus of each province, the National Bureau of Statistics, the official website of the General Administration of Sport of China, the China Statistical Yearbook, the China Rural Statistical Yearbook, the China Taobao Rural Research Report, and the China Research Data Service Platform (CNRDS). Rural e-commerce activity is measured using provincial-level indicators, while the digital financial inclusion index is derived from the Peking University Digital Financial Inclusion Index.

### 3.2 Construction of empirical model

To verify H1, Model 1 was constructed to examine the impact of digital countryside construction (DCC) on the development of the sports industry (Sic*_it_*). This model also incorporated various selected control variables, such as government intervention (Gov*_it_*), the level of financial development (Fin*_it_*), farmers’ investment capacity (Fic*_it_*), the extent of rural road governance (Roa*_it_*), and the level of education (Edu*_it_*). The regression coefficients of these variables were also introduced, denoted by ${\left\{\alpha_{j}\right\}}_{j=1}^{6}$ . If H1 holds, the regression coefficient of DCC (*α*_1_) is expected to be significantly greater than 0, indicating that *Sic* promotion is greater than 0. This confirms that DCC fosters the development of the sports industry. Additionally, Year*_t_* represents the fixed temporal effect, while City*_t_* dneotes the fixed regional effect. These variables are used to control for the common shock of unobservable temporal and regional factors on the development of the sports industry, and *ε* is a random error term. Therefore, Sic*_it_* is calculated as:


(7) $$\begin{array}{c} {\mathrm{Sic}}_{\mathrm{it}}=\alpha_{0}+\alpha_{1}{\mathrm{DCC}}_{\mathrm{it}}+\alpha_{2}{\mathrm{Gov}}_{\mathrm{it}}+\alpha_{3}{\mathrm{Fin}}_{\mathrm{it}}+\alpha_{4}{\mathrm{Fic}}_{\mathrm{it}}\\ +\alpha_{5}{\mathrm{Roa}}_{\mathrm{it}}+\alpha_{6}{\mathrm{Edu}}_{\mathrm{it}}+{\text{Year}}_{t}+{\text{City}}_{i}+\varepsilon \end{array}$$

Subsequently, Model 2 was constructed to examine the moderating effect of FMA on the relationship between DCC and the sports industry. This model highlighted the effect of the interaction between DCC and FMA on the sports industry, which is represented by DCC*_it_* × FMA*_it_*. Consequently, Sic*_it_* is calculated as:


(8) $$\begin{array}{c} {\mathrm{Sic}}_{\mathrm{it}}=\beta_{0}+\beta_{1}{\mathrm{DCC}}_{\mathrm{it}}+\beta_{2}{\mathrm{DCC}}_{\mathrm{it}}\times {\mathrm{FMA}}_{\mathrm{it}}\\ +\beta_{3}{\mathrm{FMA}}_{\mathrm{it}}+\beta_{j}{\text{control}}_{\mathrm{it}}+{\text{Year}}_{t}+{\text{City}}_{i}+\varepsilon \end{array}$$

To examine the mediating effect of rural consumption upgrading (RCU) on the relationship between DCC and the development of the sports industry, Model 3 was constructed, yielding:


(9) $${\mathrm{Sic}}_{\mathrm{it}}=\theta_{0}+\theta_{1}{\mathrm{DCC}}_{\mathrm{it}}+\theta_{j}{\text{control}}_{\mathrm{it}}+{\text{Year}}_{t}+{\text{City}}_{i}+\varepsilon$$

(10) $${\mathrm{RCU}}_{\mathrm{it}}=\theta_{0}+\theta_{1}{\mathrm{DCC}}_{\mathrm{it}}+\theta_{j}{\text{control}}_{\mathrm{it}}+{\text{Year}}_{t}+{\text{City}}_{i}+\varepsilon$$

(11) $$\begin{array}{l} {\mathrm{Sic}}_{\mathrm{it}}=\theta_{0}+\theta_{1}{\mathrm{DCC}}_{\mathrm{it}}+\theta_{2}{\mathrm{RCU}}_{\mathrm{it}}+\theta_{j}{\text{control}}_{\mathrm{it}}+\\ {\text{Year}}_{t}+{\text{City}}_{i}+\varepsilon \end{array}$$

## 4 Analysis of empirical result

### 4.1 Descriptive statistics

Descriptive statistical analysis was initially conducted to clarify the variation characteristics of each variable and achieve a more comprehensive understanding of the samples’ development patterns. To mitigate potential heteroskedasticity in empirical regression, natural logarithmic transformation was applied to the following variables: financial development, rural household investment capacity, rural road governance level, education level, and RCU. As shown in Table 3, the mean and standard deviation for DCC were 0.1122 and 0.0963, respectively, whereas those for the sports industry were 0.1182 and 0.0943. These values suggest a relatively low variability and a concentrated distribution across samples. Subsequent analysis of the standard deviation, maximum, and minimum values for other variables reveals considerable variations in financial development, rural investment capacity, and road governance levels across different provinces. Regional disparities in economic policy, geographic coverage, and resource endowments likely explain these discrepancies.

**Table 3:** Descriptive statistics analysis.

Variable name	Observations	Standard deviation	Minimum value	Maximum value	Mean
Digital countryside construction	189	0.0963	0.0023	0.6856	0.1122
Sports industry	189	0.0943	0.0003	0.6138	0.1182
Government intervention	189	0.0588	0.0823	0.3737	0.2006
The level of financial development	189	0.4995	0.291	3.379	1.541
The investment capacity of rural households	189	0.5793	0.1035	3.1748	2.3249
The governance level of rural roads	189	0.2935	0.7055	1.9615	1.4462
Education level	189	0.1423	3.0103	3.884	3.4252
Factor market-oriented allocation	189	0.0885	0.0027	0.671	0.0958
Rural consumption upgrading	189	0.1656	3.3101	3.9809	3.7052

### 4.2 Benchmark regression analysis of DCC and the sports industry development

To account for individual regional and temporal fixed effects, a panel two-way fixed-effects model (Equation 7) was employed, with the growth of the sports industry serving as the dependent variable and DCC as the key independent variable. Government intervention, financial development, rural household investment capacity, road governance level, and education level were included as control variables. The regression results are presented in Table 4.

**Table 4:** Benchmark regression model.

Primary indicator	*Sic* (1)	*Sic* (2)	*Sic* (3)
DCC	0.3359***	0.3791***	0.3129***
	5.59	7.52	5.49
Gov		−0.0479	−0.1031*
		−0.78	−1.69
Fin		0.0275***	0.0411***
		2.97	4.66
Fic		0.0252***	0.0218***
		3.22	2.67
Roa		−0.0139	−0.0128
		−0.89	−0.80
Edu		0.2443	0.0505
		0.71	1.28
Year	YES	NO	YES
City	YES	NO	YES
R^2^	0.5023	0.5314	0.4901
F	10.45***	13.59***	9.72***
N	189	189	189

t-statistics in parentheses, ***, **, and * indicate 1, 5, and 10% significance levels, respectively, below.

Model *Sic* (1) presents the baseline regression without control variables. Model *Sic* (2) includes control variables but omits fixed effects, while Model *Sic* (3) incorporates both control variables and regional and temporal fixed effects. In all three models, the regression coefficients for digital countryside construction (DCC) are 0.3359, 0.3791, and 0.3129, respectively—all of which are positive and statistically significant at the 1% level. This suggests that digital countryside construction significantly promotes the development of the sports industry.

The regression results in Table 4 indicated that enhanced digital infrastructure provided an essential foundation for connecting rural sports resources. The proliferation of online platforms and social media has reduced promotional costs, broadened market access for rural sports brands, and enhanced accessibility and participation in sports consumption for rural residents (Fu, 2024). Meanwhile, digital governance tools have assisted planning and performance assessment, enabling governments to adjust policies based on real-time data. This further enhances investment efficiency and fosters cross-sector integration through initiatives such as “Internet + Sports + Tourism” and “E-commerce + Rural Sports Equipment (Zhang et al., 2025).” This multi-dimensional integration, centered on sports events, drives the coordinated development of related sectors such as leisure, recreation, and tourism (Zhang et al., 2025).

However, there remains a paucity of research on the nexus between DCC and the growth of the sports industry in China. International literature suggests that the digital economy plays a significant role in facilitating the expansion of scale and diversification of services within the urban sports industry by enhancing resource allocation efficiency and reducing information asymmetry (Li and Han, 2023; Krishna et al., 2024). This study further indicates that such a mechanism may also be operative in rural contexts. Particularly against the backdrop of a national strategic emphasis on synergistically advancing “digital villages” and “mass fitness,” the catalytic role of digitalization in promoting the rural sports industry assumes heightened policy relevance and practical significance.

Regarding the control variables, Models *Sic* (2) and *Sic* (3) provide the following insights: Government intervention exhibits negative coefficients of −0.0479 and −0.1031, respectively. While not significant without fixed effects, the coefficient becomes significant at the 10% level when fixed effects are included, indicating that structural differences across regions and time periods highlight the negative influence of government intervention. Financial development shows positive coefficients of 0.0275 and 0.0411, both significant at the 1% level, suggesting that financial advancement supports optimized capital allocation, DCCersified financing, and reduced costs, thereby facilitating investment and innovation in the sports industry. The investment capacity of rural households also has a significant positive impact, indicating that increased capital supports infrastructure development and boosts local sports consumption, thus stimulating the synergistic growth of the sports industry chain (Liu et al., 2024). However, road governance and education levels do not exhibit statistically significant effects in this analysis, implying their influence may be limited or overshadowed by more dominant factors within this economic structure.

### 4.3 Endogeneity test

#### 4.3.1 Endogeneity test

When assessing the impact of DCC on the development of the sports industry, two potential key sources of endogeneity arise. The first is the omitted variable bias, where unobservable factors—such as local policy initiatives or regional cultural attributes—might simultaneously influence the development of both the digital infrastructure and the sports industry growth, leading to biased regression estimates. The second is the issue of reverse causality: digital technologies promote the dissemination and interactivity of sports events, thereby fostering the development of the sports industry. Consequently, the growth of the sports industry may further drive the innovation and application of digital technologies in rural construction. This dynamic, bidirectional relationship complicates the establishment of a clear causal order, reflecting a mutually reinforcing feedback loop between technological innovation and industrial development.

To address this endogeneity problem, this study uses the lagged one-period value of digital countryside construction (DCC-IV) as an instrumental variable. The results from the two-stage least squares (2SLS) regression are presented in Table 5. In the first stage, the F-statistic of 74.19 indicates that the instrumental variable has strong explanatory power for the endogenous regressor. Furthermore, the Cragg-Donald Wald F-statistic exceeds the 10% Stock-Yogo critical threshold of 16.38, confirming the absence of a weak instrument problem. In the second stage, the estimated coefficients remain positive and statistically significant at the 1% level, consistent with the baseline model results. These findings validate the robustness of the study’s conclusions and affirm the significant positive impact of digital countryside construction on the development of the sports industry.

**Table 5:** Results of endogeneity test.

Primary indicator	*Sic*(4)	
	Phase I	Phase II
DCC		0.9157^***^
		(13.12)
DCC-IV	0.7532^***^	
	(26.95)	
Control variable	Yes	Yes
Year	Yes	Yes
City	Yes	Yes
R^2^	0.8599	0.5782
Phase I F-value	74.19^***^	
Chi2		216.91^***^[16.38]
N	168	168

Values in parentheses are Stock-Yogo test thresholds at the 10% level.

#### 4.3.2 Its robustness test

##### 4.3.2.1 Tailoring for outliers

To ensure the robustness of the baseline regression results and to minimize the influence of outliers, the top and bottom 1% of the data values for each variable were winsorized. After this trimming process, the adjusted variables were re-substituted into the regression model for verification. The results, presented in Model *Sic* (5) of Table 6, show that the coefficient for digital countryside construction remains statistically significant at the 1% level. This confirms the robustness of the estimated positive impact on the development of the sports industry.

**Table 6:** Robustness test results.

Primary indicator	*Sic* (5)	*Sic* (6)	*Sic* (7)
*Nqu*	0.2775^***^	0.3768^***^	
	(2.19)	(2.43)	
Mp			0.7753^***^
			(3.20)
Control variable	Yes	Yes	Yes
Year	Yes	Yes	Yes
City	Yes	Yes	Yes
R^2^	0.5182	0.5612	0.4367
F	18.72^***^	46.91^***^	6.76^***^
N	189	153	189

##### 4.3.2.2 Exclusion of municipalities directly under central government

Given that municipalities directly under the central government—Beijing, Tianjin, Shanghai, and Chongqing—differ substantially from other provincial units in terms of economic structure, policy environment, and data characteristics, their inclusion may distort the regression results. Therefore, these four municipalities were excluded, and the model was re-estimated. The findings, reported in Model *Sic* (6) of Table 5, indicate that the empowering effect of digital countryside construction on the sports industry remains statistically significant. This further supports the reliability and generalizability of the baseline conclusions.

##### 4.3.2.3 Replacement of explanatory variables

To avoid bias arising from the construction of the digital rural evaluation index system, this study follows the approach of Lei et al. (2023) et al. and uses the number of mobile phones owned by rural residents per 100 households (Mp) as a proxy variable for digital countryside construction in the robustness test. The specific results are presented in Column (7) of Table 6. The regression coefficient is 0.7753 and is significant at the 1% level, indicating that the empirical results are robust.

### 4.4 Mechanism analysis

#### 4.4.1 Moderating effect analysis of MFA

Moderating effect analysis examines whether and how the strength or direction of a relationship between two variables changes under different conditions, thereby increasing the explanatory power of the model. Specifically, it examines whether the factor marketization configuration moderates the effect of DCC on the development of the sports industry was examined. This was conducted using Equation 8, with the coefficients of the interaction terms between DCC and factor marketization configuration being the primary focus. As illustrated in Table 7, the interaction terms remained significantly positive at the 10% level, suggesting that factor marketization exert a positive moderating effect. This demonstrates that MFA enhances the positive effect of DCC on the development of the sports industry.

**Table 7:** Results of moderating effects analysis.

Primary indicator	*Sic* (8)
DCC	0.2471^***^
	(3.05)
DCC x FMA	0.9738^*^
	(1.75)
Control variable	Yes
Year	Yes
City	Yes
R^2^	0.5064
F	27.92^***^
N	189

Specifically, MFA enhances the mobility and allocation of market resources, such as land, labor, and capital, thereby boosting the efficiency of resource distribution. This forges a robust institutional and economic foundation for the integration of digital technologies in rural areas (Liu et al., 2025). Furthermore, market-oriented reforms increase the adaptability and responsiveness of the sports industry to the requirements of DCC. Factor-efficient markets also help reduce systemic transaction costs, incentivize private capital to engage in the rural sports sector, and support the upgrading of the sports product supply structure and service quality (Ma and Huang, 2024). Consequently, MFA not only strengthens the economic foundations of DCC but also fosters the high-quality development of the sports industry.

#### 4.4.2 Mediating effect analysis of RCU

Mediating effect analysis investigates the mechanism through which an independent variable indirectly affects a dependent variable via a mediating variable, thereby deepening the understanding of the internal relationship among variables. Herein, the mediating role of RCU in the relationship between DCC and the development of the sports industry was examined using a three-stage regression analysis, as described by Equations 9–11. As depicted in Table 8, the regression coefficients of DCC in models *Sic* (9) and *Sic* (10), as well as the regression coefficients of RCU in models Rc (1) and *Sic* (10), all exhibited significantly positive values. This indicates that DCC exerts a significant positive effect not only on RCU but also on the development of the sports industry. Moreover, the regression coefficient of DCC decreased from 0.3129 in *Sic* (9) to 0.2959 in *Sic* (10) upon the inclusion of the mediating variable, suggesting the a partial mediating effect. This confirms that DCC fosters the development of the sports industry in part through the pathway of RCU.

**Table 8:** Results of the mediating effect analysis.

Primary indicator	*Sic* (9)	*Rc* (1)	*Sic* (10)
DCC	0.3129^***^	0.0476^**^	0.2959^***^
	(3.20)	(2.30)	(5.13)
Rc			0.3578^*^
			(1.62)
Control variable	Yes	Yes	Yes
Year	Yes	Yes	Yes
City	Yes	Yes	Yes
R^2^	0.5064	0.8669	0.5520
F	27.92^***^	678.42^***^	9.34^***^
N	189	189	189

Specifically, the deep integration of digital technologies in rural areas has improved information access, gradually reshaped consumption attitudes, and markedly enhanced the awareness of sports consumption among rural residents. Consequently, rural residents have shifted their survival-oriented consumption pattern to one that is developmental and enjoyment-based (Yang and Li, 2025). Furthermore, digital technologies have improved the accessibility and convenience of sports products and services, broadened the market reach of the sports industry, and reinforced the role of RCU in driving and supporting the growth of the sports industry. Therefore, DCC indirectly facilitates the high-quality development of the sports industry.

### 4.5 Structural equation modeling

#### 4.5.1 Structural equation modeling analysis

In this study, Smart PLE software is utilized for PLS-SEM modeling, enabling the construction of both reflective and formative measurement models (Kono and Sato, 2022). Additionally, PLS-SEM can construct a hybrid model that combines both approaches, facilitating improved validation. As shown in Table 9, the R^2^ coefficient reflects the combined effect of exogenous latent variables on endogenous latent variables. In this study, the R^2^ value for the endogenous variable Rural Consumption Upgrading is 0.235, indicating a weak model, whereas the R^2^ value for the sports industry is 0.800, indicating a strong model. Furthermore, Q^2^ is used to assess the model’s predictive relevance. The Q^2^ value for the sports industry in this study is 0.576, indicating that the model demonstrates strong predictive ability (Khan et al., 2019). Finally, the model’s fit was assessed using the Standardized Root Mean Square Residuals (SRMR), with the SRMR value for the estimated model being 0.036, satisfying the model fit requirement (Garson, 2019).

**Table 9:** The values of R^2^, Q^2^.

Constructs	R^2^	Remarks	Q^2^	Predictive relevance	Remarks
Rural consumption upgrading	0.235	Moderate	0.177	Q^2^ > 0	Moderate
Sports industry	0.8	Weak	0.576	Q^2^ > 0	Strong

#### 4.5.2 Research hypothesis testing

This study measured the variance inflation factors (VIF) of all latent variables in the inner model. The VIF values among all latent variables in the research model ranged from 1.153 to 3.175 (see Table 10), indicating the absence of common method bias. Secondly, ƒ^2^ denotes the effect size, which is used to evaluate whether the omitted constructs have a substantial impact on the endogenous constructs (Shintaro and Mikihiro, 2022). In this study, the ƒ^2^ values of the exogenous variables—digital countryside construction, factor marketized allocation, and their interaction term—on the endogenous variables (rural consumption upgrading and the sports industry) all exceed 0.15 (see Table 10), indicating that these exogenous variables have more than a moderate explanatory effect on the corresponding endogenous variables, meeting the threshold for substantial influence (Wassertheil, 1970). This suggests that each exogenous variable exerts a meaningful influence on its respective endogenous variable and should not be excluded from the model.

**Table 10:** Results of hypotheses testing.

Hypotheses	Original sample (β)	Standard deviation	T statistics	*p* values	Support	VIF	F2
Main model							
digital countryside construction → Sports industry	0.563^***^	0.092	6.107	0	Yes	1.46	1.088
Rural consumption upgrading → Sports industry	0.362^***^	0.071	5.11	0	Yes	1.39	0.471
Digital countryside construction → Rural consumption upgrading	0.472^***^	0.049	9.671	0	Yes	1.153	0.253
Mediating effect of rural consumption upgrading							
Digital countryside construction → Rural consumption upgrading → Sports industry	0.171^***^	0.038	4.497	0	Yes	--	--
Moderating effect of factor market-oriented allocation							
Factor market-oriented allocation x Digital countryside construction → Sports industry	0.522^***^	0.119	4.376	0	Yes	3.087	0.296

**Figure 2:**
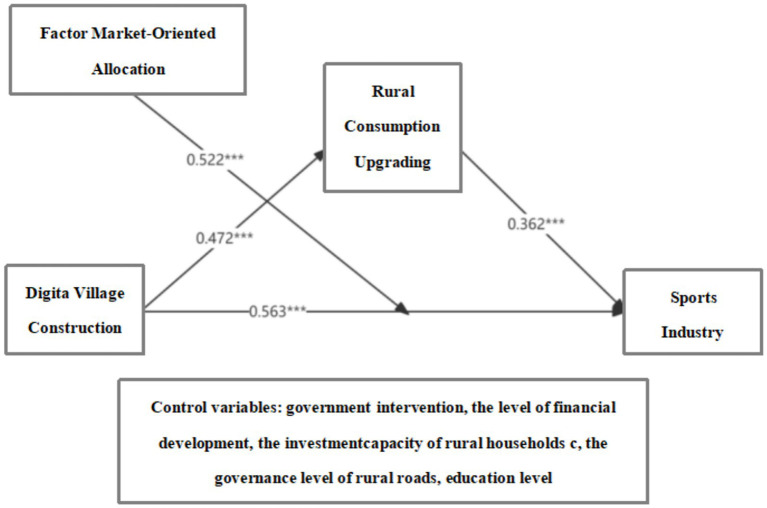
Diagram of the path model for the research hypothesis.

In summary, this study analyzes the mechanisms through which digital countryside construction, rural consumption upgrading, and factor market-oriented allocation influence the development of the sports industry using PLS-SEM, while controlling for government intervention, the level of financial development, the investment capacity of rural households, the governance level of rural roads, education level. The final model diagram is presented in Figure 2.

The results indicate that all structural paths are statistically significant, and the model demonstrates robustness (Figure 2) digital countryside construction exerts a significant positive effect on the sports industry (*β* = 0.563, *p* < 0.001, f^2^ = 1.088), making it the most critical direct influencing factor; it also indirectly promotes the development of the sports industry through rural consumption upgrading, which exhibits a significant partial mediation effect (β = 0.171, *p* < 0.001). Rural consumption upgrading also exerts a significant positive effect on the sports industry (β = 0.362, *p* < 0.001, f^2^ = 0.471). In addition, the moderating effect analysis reveals that factor market-oriented allocation significantly enhances the effect of digital countryside construction on the sports industry (β = 0.522, *p* < 0.001, f^2^ = 0.296), suggesting that the institutional environment plays a crucial role in the effectiveness of policy implementation. This finding confirms that the market-oriented allocation of factors has a positive moderating effect on the relationship between digital countryside construction and the sports industry. In summary, the study elucidates both the direct and indirect pathways through which digital countryside construction promotes the development of the sports industry, while highlighting the synergistic effects of rural consumption upgrading and the market-oriented institutional environment. Accordingly, the research hypotheses H1, H2, and H3 are further validated through PLS-SEM analysis.

### 4.6 Heterogeneity analysis

Given the substantial variations in natural conditions, economic resources, and development levels across different regions in China, a heterogeneity analysis was subsequently conducted, focusing on the regional divisions established by the National Bureau of Statistics. To examine the differential impact of DCC on the development of the sports industry across the entire country, the samples were classified into three primary regions: eastern China (including Beijing, Tianjin, Hebei, Liaoning, Shanghai, Jiangsu, Zhejiang, Fujian, Shandong, and Guangdong), central China (including Shanxi, Inner Mongolia, Anhui, Jiangxi, Henan, Hubei, and Hunan), and western China (including Chongqing, Sichuan, Guizhou, and Yunnan).

As shown in Table 11, the impact of DCC on the development of the sports industry exhibited pronounced regional heterogeneity. The regression results demonstrated a significant positive effect in eastern China, a negative but statistically insignificant effect in central China, and a positive but also insignificant effect in western China. These disparities can be attributed to structural differences among different regions, including economic foundations, digital infrastructure, stages of sports industry development, and policy support.

**Table 11:** Results of the heterogeneity analysis.

Primary indicator	*Sic* (11)	*Sic* (12)	*Sic* (13)
	(Eastern)	(Central)	(Western)
DCC	0.2742^***^	−0.0032	0.2732
	(3.15)	(2.07)	(1.61)
Control variable	Yes	Yes	Yes
Year	Yes	Yes	Yes
City	Yes	Yes	Yes
R^2^	0.4580	0.2220	0.5749
F	6.85^***^	3.12^***^	1.62^***^
N	90	63	36

Eastern China is characterized by a higher level of economic development, greater penetration of digital technology, and deeper urban–rural integration, thereby offering a favorable market environment and technological support for the growth of the sports industry.

In central China, the impact of DCC on sports industry development has been relatively limited. The reasons are twofold: Firstly, compared to eastern China, the central region may experience delays in policy coordination between DCC and sports industry development. Certain policies have not successfully integrated digital technologies into the local sports industry development plans, preventing policy benefits from creating synergies and impeding industrial advancement. Secondly, residents in central China may still favor traditional sports consumption habits, exhibiting relatively lower acceptance of digital sports services. Despite established digital infrastructure, the inertia of consumer preferences and habits hinders the potential simulative effect of digital technologies on sports consumption.

Regarding western China, the impact of DCC on sports industry development is minimal, or even negative, likely due to the following reasons: Firstly, considering the limited public resources in western China, substantial investments on digital infrastructure could temporarily divert critical resources from other sectors crucial to sports industry development, causing the “crowding-out effect” of infrastructure investments. This trade-off dynamic may explain why initial investments in DCC have failed to yield the anticipated positive effects. Secondly, given the significant geographical constraints, educational disparities, and digital literacy gaps in western China, it would take considerable time for the implementation of DCC to bridge the ever-expanding digital divide between urban and rural residents. Thirdly, compared to the eastern and central regions of China, the sports industry in the western part is underdeveloped, characterized by a low level of marketization and incomplete industrial chains. In this context, digital technology alone may find it challenging to generate a significant impact.

## 5 Conclusion

Herein, the impact of DCC on the development of the sports industry was investigated utilizing panel data from 21 provinces in China spanning from 2015 to 2023. Through the application of fixed effects models, moderating effect models, and mediating effect models, the mechanisms and pathways by which DCC influences sports industry development were comprehensively examined. The following conclusions can be drawn:

The present study offers significant theoretical contributions to the existing literature in the fields of digital economy and industrial development. Firstly, it systematically explored the intrinsic logical relationship between comprehensive DCC and sports industry development, bridging a research gap in this field. Unlike previous studies that have concentrated on individual components such as the digital economy or digital finance (Deng and Li, 2023), the present study approached DCC as a comprehensive strategy, revealing its multifaceted impact on the development of a particular industry. Secondly, by constructing moderation and mediation models, the present study made the first attempt to quantify the transmission mechanisms of MFA and RCU within this influence chain, deepening the understanding of how digital strategies drive industrial development. Thirdly, the heterogeneity analysis not only confirmed the regionally varied impact of DCC on sports industry development but also offered preliminary theoretical insights into why this impact was less pronounced in central and western China, forging a hypothesis foundation for future in-depth regional studies.

The findings provide essential practical insights for the synergistic advancement of DCC and sports industry development. Firstly, policy coordination and the enhancement of “software investments” should be strengthened. The driving impact of DCC on the development of the sports industry does not merely stem from “hardware investments,” such as those in digital infrastructure, but rather involves “software investments,” which warrant greater emphasis. For instance, policy guidance and financial support should be introduced to encourage the development of digital sports service platforms and innovative, market-oriented operational mechanisms. This will help ensure that digital accomplishments are effectively translated into key drivers of industrial development (Ye and Chen, 2023). Secondly, region-specific development strategies and measures, tailored to local conditions, should be implemented. The heterogeneity analysis presented herein revealed the distinct responses to DCC across different regions, suggesting that provincial governments should implement differentiated development strategies rather than adopting a one-size-fits-all approach. Furthermore, market entities should be encouraged to actively engage in digital transformation (Wang and Huang, 2025). Specifically, within the sports industry, sports companies and other relevant market participants should take the initiative to embrace digital transformation. This will enable market entities to capitalize on opportunities presented by DCC, explore novel business models, and identify promising growth areas.

The findings of the present study hold significant practical and theoretical implications, not only for China but also offering valuable insights for other nations navigating digital transformation and sports industry development. Many countries, especially those that are developing, encounter similar challenges in advancing digital transformation, particularly in ensuring that “hardware investments” effectively translate into “soft drivers” for industrial growth. For instance, India is actively promoting its “digital India” strategy to increase the penetration of information technology in rural areas; however, the development of its sports industry is hindered by bottlenecks in funding, talent, and business model innovation (Singh and Nimkar, 2020). Similarly, Brazil is accelerating internet access in rural regions, yet its sports industry heavily depends on major events, such as soccer, and is deficient of effective integration with the local digital technology ecosystem (Hall and Nicholas, 2019). In contrast, China possesses exceptional advantages in terms of top-level design and policy coordination. Its DCC strategy facilitates systematic planning across multiple dimensions: infrastructure, digital services, and industrial development; however, China still faces challenges associated with regional heterogeneity and policy implementation, as revealed herein. These findings offer valuable insights for other countries developing comparable strategies.

## 6 Research limitations and future directions

The present study sheds lights on the relationship between DCC and the development of the sports industry. However, limitations persist in several aspects, including reliance on macro-level data, the construction of indicator system, and potential endogeneity issues. Specifically, due to the use of provincial-level panel data, the present study does not capture detailed impact mechanisms at the municipal, county, or micro-level entities (Özmen et al., 2016). Additionally, limitations within official statistical definitions and data availability may have excluded certain emerging or informal business models from the evaluation framework (Kropat et al., 2017; Taylan et al., 2010; Semih et al., 2022). While econometric methods were employed to account for these factors, the potential bidirectional causal relationship between them warrants further validation through more sophisticated models in future research.

To address these limitations, future research endeavors can be made in the following directions: Firstly, employing finer-grained microdata could enable deeper exploration of the specific impact mechanisms of DCC at the enterprise and household levels (Roy and Bhaumik, 2018; Babaee et al., 2021). Secondly, incorporating additional potential mediating and moderating variables—such as talent, finance, and cultural factors—could reveal more comprehensive impact pathways (Savku, 2023). Ultimately, cross-national comparative analysis should be conducted to examine the universality and differences concerning the impact of digital strategies on sports industry development across diverse institutional and cultural contexts, thereby providing more universally applicable theoretical guidance for global policy formulation.

## Data Availability

The raw data supporting the conclusions of this article will be made available by the authors, without undue reservation.
